# Role of insulin signaling impairment, adiponectin and dyslipidemia in peripheral and central neuropathy in mice

**DOI:** 10.1242/dmm.015750

**Published:** 2014-04-24

**Authors:** Nicholas J. Anderson, Matthew R. King, Lina Delbruck, Corinne G. Jolivalt

**Affiliations:** School of Medicine, Department of Pathology, University of California San Diego, 9500 Gilman Drive, La Jolla, CA 92093, USA.

**Keywords:** Glucose, High-fat diet, Insulin, Neuropathy

## Abstract

One of the tissues or organs affected by diabetes is the nervous system, predominantly the peripheral system (peripheral polyneuropathy and/or painful peripheral neuropathy) but also the central system with impaired learning, memory and mental flexibility. The aim of this study was to test the hypothesis that the pre-diabetic or diabetic condition caused by a high-fat diet (HFD) can damage both the peripheral and central nervous systems. Groups of C57BL6 and Swiss Webster mice were fed a diet containing 60% fat for 8 months and compared to control and streptozotocin (STZ)-induced diabetic groups that were fed a standard diet containing 10% fat. Aspects of peripheral nerve function (conduction velocity, thermal sensitivity) and central nervous system function (learning ability, memory) were measured at assorted times during the study. Both strains of mice on HFD developed impaired glucose tolerance, indicative of insulin resistance, but only the C57BL6 mice showed statistically significant hyperglycemia. STZ-diabetic C57BL6 mice developed learning deficits in the Barnes maze after 8 weeks of diabetes, whereas neither C57BL6 nor Swiss Webster mice fed a HFD showed signs of defects at that time point. By 6 months on HFD, Swiss Webster mice developed learning and memory deficits in the Barnes maze test, whereas their peripheral nervous system remained normal. In contrast, C57BL6 mice fed the HFD developed peripheral nerve dysfunction, as indicated by nerve conduction slowing and thermal hyperalgesia, but showed normal learning and memory functions. Our data indicate that STZ-induced diabetes or a HFD can damage both peripheral and central nervous systems, but learning deficits develop more rapidly in insulin-deficient than in insulin-resistant conditions and only in Swiss Webster mice. In addition to insulin impairment, dyslipidemia or adiponectinemia might determine the neuropathy phenotype.

## INTRODUCTION

Diabetes mellitus affects 8% of the US population and is sub-classified into type 1 (insulin-deficient) and type 2 (insulin-resistant) diabetes. Peripheral neuropathy is the most common of the complications associated with long-term diabetes mellitus and develops in more than half of all diabetic patients (Calcutt et al., 2009). Peripheral neuropathy affects all peripheral nerves and results in sensory loss, pain and autonomic dysfunction that severely reduces the quality of life of type 1 and type 2 diabetic patients. In addition to glucotoxicity as a primary pathogenic mechanism (Gabbay, 1973), impaired insulin and growth factor signaling are emerging as glucose-independent pathogenic mechanisms of diabetic neuropathy. Several studies have also demonstrated a coincidence between diabetic complications and impaired function of the central nervous system (CNS) (Ferguson et al., 2003; Ryan and Williams, 1993), suggesting that the brain is susceptible to the same processes that underlie other complications of diabetes. Studies have found a relative risk of about 1.9 to 2.3 of developing Alzheimer’s disease (AD) for individuals with diabetes (Leibson et al., 1997; Ott et al., 1996; Ronnemaa et al., 2009), and this risk can increase up to 5.5 in the presence of ApoE4, a risk factor for AD (Peila et al., 2002). Most recently, diabetes was shown to increase not only the risk of dementia but also the risk of progression from mild cognitive impairment to AD (Velayudhan et al., 2010). Encephalopathy, defined as electrophysiological and structural disturbances in the brain associated with cognitive deficits, occurs in both type 1 and type 2 diabetic subjects (Biessels et al., 2008, Cukierman et al., 2005; Desrocher and Rovet, 2004; Manschot et al., 2006; McCarthy et al., 2002; Reaven et al., 1990; Ryan et al., 1985; Ryan and Williams, 1993). Uncontrolled diabetes (no insulin treatment) was associated with the development of AD, whereas individuals with controlled diabetes showed no increased dementia, suggesting a role of impaired insulin signaling in the development of neurodegeneration and AD (Xu et al., 2009). In parallel, the insulin-signaling pathway was shown to be impaired in the brain of individuals with AD (Revill et al., 2006).

The pathological mechanisms leading to damage of the peripheral nervous system (PNS) and CNS are not yet well understood, impeding the development of therapies. Although mechanisms are unclear, disruption of insulin signaling is strongly suggested, in particular insulin resistance or deficiency in the brain (Craft, 2005). In this study, we compared the development of PNS and CNS neuropathy in two strains of mice with type 1 insulin-deficient or type 2/obesity insulin-resistant diabetes or pre-diabetes and found that, independent of glucose level and in addition to the insulin signaling impairment, lipid and adiponectin profiles can determine the phenotype of the neuropathy.

## RESULTS

A summary of the results described below is provided in Table 1.

**Table 1.**
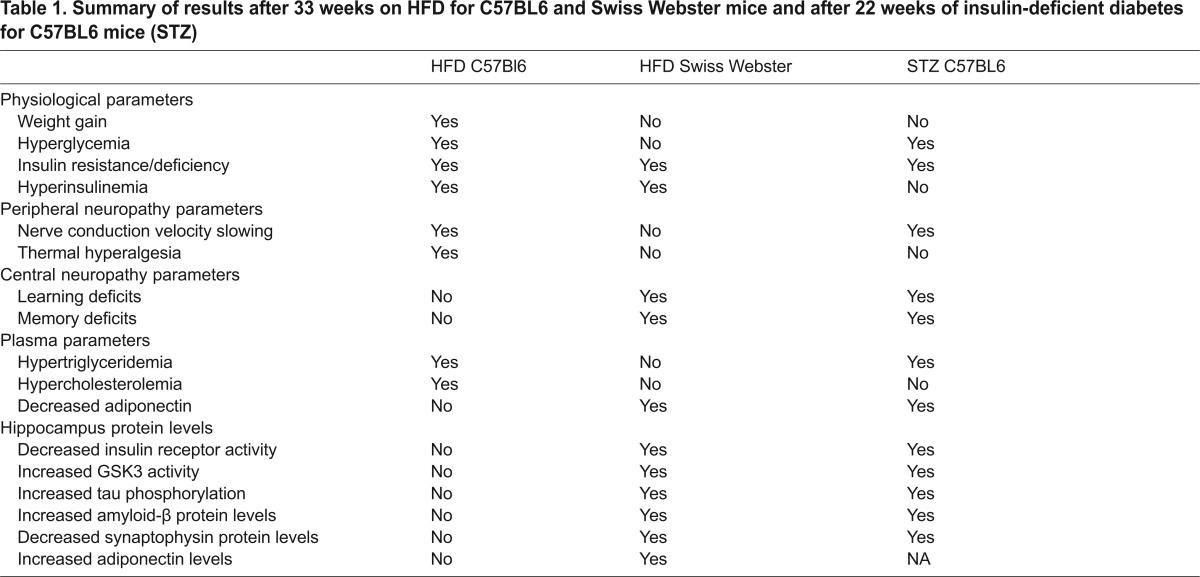
Summary of results after 33 weeks on HFD for C57BL6 and Swiss Webster mice and after 22 weeks of insulin-deficient diabetes for C57BL6 mice (STZ)

### Metabolic parameters

**Fig. 1.**
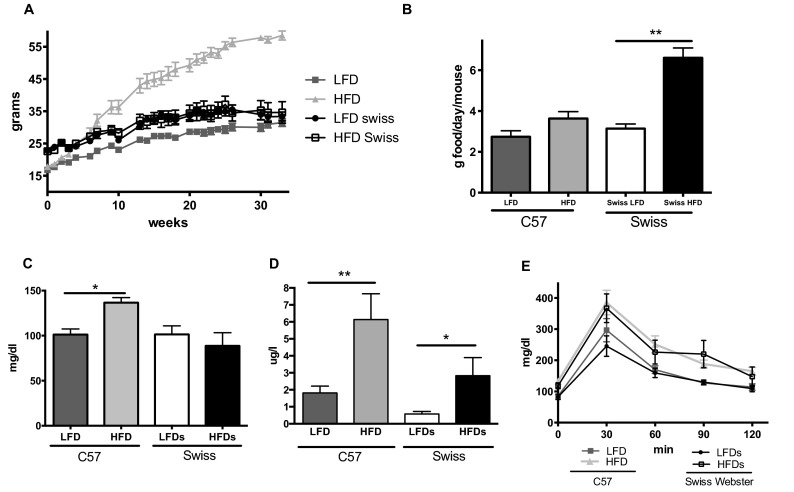
**Metabolic parameters.** (A) Body weight, (B) food consumption, (C) blood glucose levels, (D) plasma insulin levels an (E) blood glucose levels after a glucose challenge are shown for C57BL6 (C57) and Swiss Webster (Swiss) mice on a low-fat diet (LFD) or high-fat diet (HFD). *P<0.05, **P<0.01 using Student’s *t*-test against their respective LFD control.

C57BL6 (C57) mice fed a low-fat diet (LFD) gained a constant weight over 32 weeks, whereas the C57 mice fed a high-fat diet (HFD) gained a significant amount of weight, weighing up to twice as much as the LFD mice by the end of the study (Fig. 1A). Streptozotocin (STZ)-induced diabetic C57 mice maintained a healthy weight for up to 22 weeks. In contrast to C57 mice, the Swiss Webster mice on a HFD and LFD had similar body weights for the whole duration of the study (Fig. 1A), despite the food consumption of the HFD mice being double that of those on a LFD (Fig. 1B).

**TRANSLATIONAL IMPACT**

**Clinical issue**

Diabetes is a metabolic disease characterized by hyperglycemia, which occurs because of insulin deficiency (type 1 diabetes) or insulin resistance (type 2 diabetes). The majority of cases fall under the second category. Approximately 350-million people worldwide are diabetic, and the incidence is predicted to double by 2050. Among diabetes complications, peripheral neuropathy (damage to the peripheral nervous system), which occurs in over half of all cases, is the most debilitating. Diabetes also affects the central nervous system and is associated with a higher incidence of dementia. Currently available therapies focus on the treatment of symptoms rather than the underlying causes. The development of targeted therapies has been impeded because mechanisms leading to peripheral and central neuropathy have not yet been clearly elucidated and the potential roles of glucose and insulin in neurological pathogenesis remain unclear.

**Results**

In this study, the authors aimed to determine whether diabetic conditions caused by a high-fat diet (HFD) can lead to damage to the peripheral or central nervous systems. Two different mouse strains were fed with high-or low-fat diets, and compared with control mice and models in which diabetes was induced using streptozotocin (STZ). Both strains developed insulin resistance in response to the HFD. Peripheral and central nervous system functions were then examined. Damage to neuronal functions was observed in mice fed with a HFD or in association with STZ-induced diabetes. Interestingly, the different mouse strains developed either peripheral or central neuropathy. The authors show that the type of neuropathy depends on whether insulin resistance or deficiency is present and accompanied by dyslipidemia or adiponectinemia.

**Implications and future directions**

This work provides evidence that the major contributor to diabetic neuropathy is insulin impairment in association with defects in fat metabolism (lipids, adiponectin). The difference in neurological phenotypes of the different strains indicates that the genetic background determines whether the peripheral or central nervous system becomes damaged in response to HFD and the induction of diabetes. The models described in the work will facilitate further investigation of the mechanisms leading to peripheral or central neuropathy. These models could also be used to test therapeutic strategies aimed at combating the molecular triggers of diabetic neuropathy.

After 8 and 30 weeks on HFD, only C57 mice had a significant increase of fasted blood glucose (Fig. 1C). Both strains of mice developed a significant hyperinsulinemia (Fig. 1D) and insulin resistance (Fig. 1E) by 9 weeks on HFD.

### Peripheral neuropathy

**Fig. 2.**
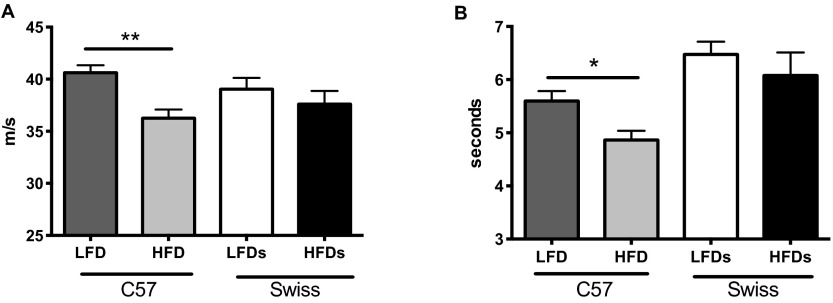
**Peripheral neuropathy parameters.** (A) Motor nerve conduction velocity and (B) thermal latencies for C57BL6 (C57) and Swiss Webster (Swiss) mice on a low-fat diet (LFD) or high-fat diet (HFD). *P<0.05, **P<0.01 using Student’s *t*-test against their respective LFD control.

Motor nerve conduction velocity (MNCV) was measured at different time points during the study. After 8 weeks, only STZ-diabetic mice had developed significantly slower MNCV (LFD_C57_: 36.1±0.9, HFD_C57_: 36.8±1.0, STZ: 31.3±0.7*, LFD_Swiss Webster_: 37.6±1.0, HFD_Swiss Webster_: 36.1±0.8 m/second, *P<0.05). By 32 weeks on HFD, only C57 mice developed a significant (*P*<0.001) slowing of MNCV (Fig. 2A). No difference was observed for Swiss Webster mice.

Thermal response of the hind paw was assessed at 8 weeks. At this time point none of the groups displayed significant changes (LFD_C57_: 6.6±0.2, HFD_C57_: 6.2±0.2, STZ: 6.1±0.4, LFD_Swiss Webster_: 7.1±0.3, HFD_Swiss Webster_: 6.6±0.4 seconds). By 32 weeks on HFD, C57 mice fed a HFD developed a significant (*P*<0.05) hyperalgesia but no difference was observed for Swiss Webster mice (Fig. 2B).

### Learning and memory deficits

To assess the changes affecting the brain, we used the Barnes maze, which has been described previously (Jolivalt et al., 2008; King et al., 2013).

**Fig. 3.**
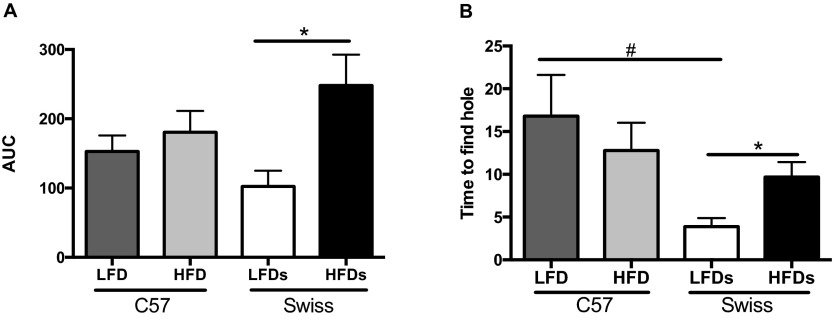
**Learning and memory behavior.** (A) Area under the curve (AUC) of the learning curves over 5 days (learning phase) and (B) time to find the hole after 3 days without exposure to the maze (memory phase) in the Barnes maze test for C57BL6 (C57) and Swiss Webster (Swiss) mice on a low-fat diet (LFD) or high-fat diet (HFD). *P<0.05, using Student’s *t*-test against their respective LFD control. ^#^*P*<0.05 using one-way ANOVA against C57BL6 LFD.

As we have previously shown for STZ-Swiss Webster mice (Jolivalt et al., 2008), STZ-C57 mice developed significant (*P*<0.05) learning impairment in the Barnes maze after 8 weeks of diabetes. Neither C57 nor Swiss Webster mice on HFD developed learning impairment at that time point [area under the curve (AUC) of 5 consecutive testing days: LFD_C57_: 225.7±35.2, HFD_C57_: 280.6±22.7, STZ: 409.5±141.7*, LFD_Swiss Webster_: 283.7±45.1, HFD_Swiss Webster_: 368.0±60.1, *P<0.05]. After 31 weeks on HFD, significant (*P*<0.05) learning impairments were detected in Swiss Webster mice (Fig. 3A). The impairments were significant by 22 weeks on HFD (data not shown). C57 mice on HFD did not develop impairments (Fig. 3A).

Memory retention was assessed after 3 days without exposure to the maze and with the escape box removed. After 8 weeks, only STZ-diabetic mice developed an impairment in locating the hole where the escape box was previously located (time to find the hole in seconds: LFD_C57_: 26.6±5.2, HFD_C57_: 18.8±2.8, STZ: 73.0±24.1, LFD_Swiss Webster_: 31.1±8.8, HFD_Swiss Webster_: 33.3±8.9). By 32 weeks, C57 mice on HFD did not show memory impairments, whereas Swiss Webster mice displayed a significant (*P*<0.05) alteration in memory retention in the Barnes maze (Fig. 3B). The basal memory ability of Swiss Webster mice on LFD was significantly (*P*<0.05) higher than that of C57 mice on LFD (Fig. 3D). The differences observed in the Barnes maze (learning and memory) for Swiss Webster mice were not due to reduced motor function, as assessed by the rotarod test (time on the accelerating rod in seconds: LFD_C57_: 27.7±2.4, HFD_C57_: 4.7±0.8*, LFD_Swiss Webster_: 26.0±2, HFD_Swiss Webster_: 21.1±4.3, *P<0.05).

### Plasma lipids

**Fig. 4.**
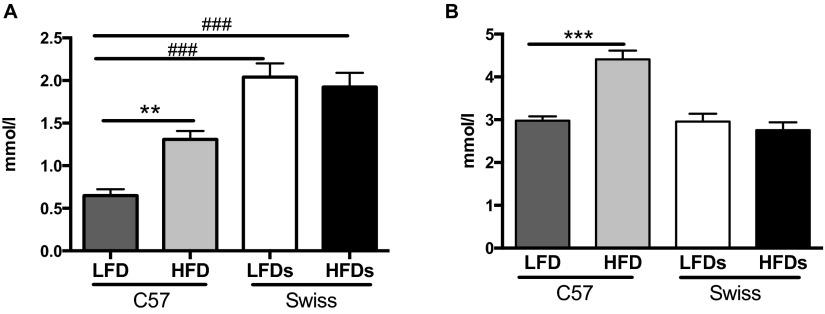
**Lipid profile.** (A) Plasma triglyceride levels and (B) plasma total cholesterol levels for C57BL6 (C57) and Swiss Webster (Swiss) mice on a low-fat diet (LFD) or high-fat diet (HFD). **P<0.01, ***P<0.001 using Student’s *t*-test against their respective LFD control. ^###^*P*<0.001 using one-way ANOVA against C57BL6 LFD.

Triglycerides (TGs), measured in terminal plasma, were significantly (*P*<0.01) increased in C57 mice fed a HFD (Fig. 4A) and in STZ-C57 mice (TG: 1.7±0.4 mmol/l) when compared with C57 mice fed a LFD. Levels of TGs in Swiss Webster mice were not different between LFD and HFD groups; however, levels for both groups were significantly (*P*<0.001) higher than those of C57 mice (Fig. 4A).

Total cholesterol levels were significantly (*P*<0.001) increased in C57 mice fed a HFD and were unchanged for Swiss Webster mice (Fig. 4B) and STZ-C57 mice (cholesterol: 2.7±0.1 mmol/l).

### Adiponectin and adiponectin receptor

**Fig. 5.**
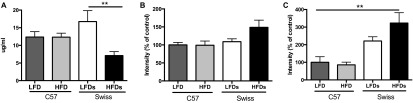
**Adiponectin profile.** (A) Plasma adiponectin levels, (B) adiponectin protein and (C) adiponectin receptor protein levels in hippocampus of C57BL6 (C57) and Swiss Webster (Swiss) mice on a low-fat diet (LFD) or high-fat diet (HFD). **P<0.01 using Student’s *t*-test against their respective LFD control.

High molecular weight (HMW) adiponectin levels were significantly (*P*<0.01) decreased in plasma from Swiss Webster mice fed a HFD compared with mice fed a LFD (Fig. 5A) and in STZ-C57 mice (7.3±1.8 μg/ml) compared with C57 fed a LFD. No difference was observed between LFD- and HFD-fed C57 mice (Fig. 5A). Adiponectin and its receptor protein levels were assessed by western blot analysis of the hippocampus. As for plasma adiponectin levels, adiponectin and adiponectin receptor protein levels were similar in C57 fed a HFD or LFD (Fig. 5B,C). In contrast, adiponectin and its receptor protein levels were increased, although not significantly (*P*<0.06), in the hippocampus of Swiss Webster mice fed a HFD (Fig. 5B,C).

### Western blot analysis

**Fig. 6.**
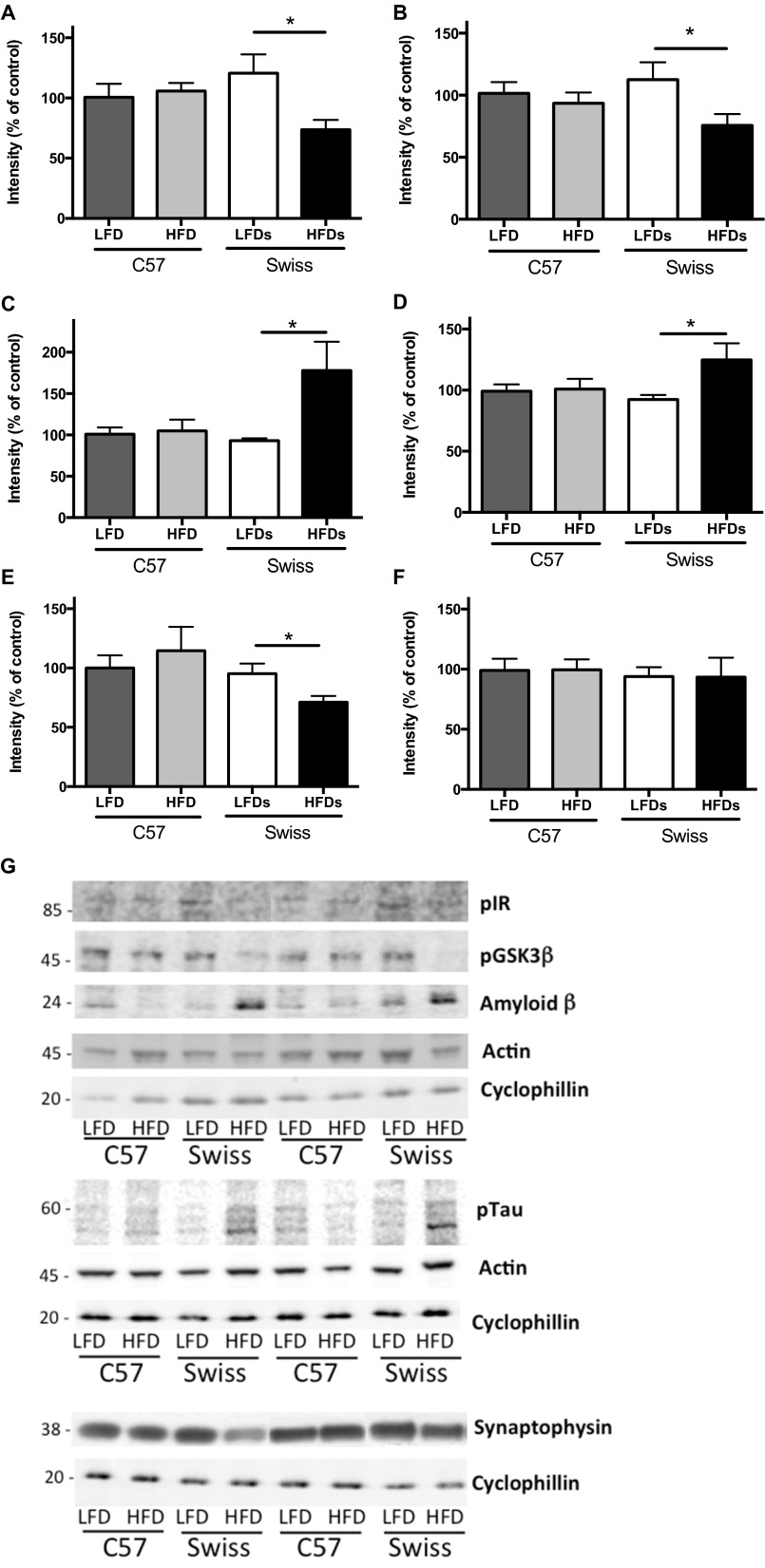
**Western blot analysis.** (A) Phosphorylated insulin receptor, (B) phosphorylated GSK3β, (C) amyloid-β (Aβ; 24 kDa), (D) phosphorylated tau, (E) synaptophysin and (F) actin protein levels normalized against cyclophilin B in the hippocampus of C57BL6 (C57) and Swiss Webster (Swiss) mice on a low-fat diet (LFD) or high-fat diet (HFD). *P<0.05, using Student’s *t*-test against their respective LFD control. (G) Representative western blot images for the proteins in A to F.

For consistency, all protein levels were normalized against the protein cyclophilin B. Cyclophilin B (20 kDa) was selected because of resolution conflicts between actin (43 kDa), usually used as housekeeping protein, and synaptophysin (38 kDa) owing to the proximity of the bands of the two proteins. However, when possible, both actin and cyclophilin B levels were assessed and the levels of both proteins were similar in each group (Fig. 6F,G).

Phosphorylation levels of insulin receptor were significantly (*P*<0.05) reduced in the hippocampus of Swiss Webster mice fed a HFD compared with mice fed a LFD and were unchanged in both groups of C57 mice (Fig. 6A,G). Similarly, phosphorylation (Ser9) levels of GSK3β were significantly (*P*<0.05) reduced, corresponding to an increased activity of GSK3, in the hippocampus of Swiss Webster mice fed a HFD compared with mice fed a LFD and were unchanged in both groups of C57 mice (Fig. 6B,G).

Levels of amyloid-β (Aβ) and phosphorylated (Ser202) tau were significantly (*P*<0.05) increased in the hippocampus of Swiss Webster mice fed a HFD compared with mice fed a LFD and were unchanged in both groups of C57 mice (Fig. 6C,D,G).

Synaptophysin levels were significantly (*P*<0.05) reduced in the hippocampus of Swiss Webster mice fed a HFD compared with mice fed a LFD and were unchanged in both groups of C57 mice (Fig. 6E,G).

## DISCUSSION

In this study, we have shown that HFD causes type 2 diabetes in C57 mice, with obesity, insulin resistance, mild hyperglycemia and peripheral neuropathy but no damage to the CNS. In Swiss Webster mice, HFD causes metabolic syndrome (pre-diabetes), with insulin resistance but normal body weight and blood glucose. In these mice, the PNS was spared but the CNS was affected. The strain dependence of the consequences of a HFD suggests that a genetic component to fat metabolism in addition to an insulin-signaling defect dictate whether the PNS or CNS is damaged.

Diabetes, type 1 and type 2, impacts the nervous system both peripherally and centrally (Lee-Kubli et al., 2014). In addition to aging, it is now accepted that diabetes is a relevant risk factor for developing dementia and AD (Craft, 2009). However, the debate about the role of insulin or glucose in the etiology of diabetes-associated dementia is growing strong. Differentiating the role of glucose from that of insulin is a difficult task in human as well as in animal research. Recently, Crane et al. (Crane et al., 2013) showed, in a longitudinal study involving more than 200 individuals, that higher blood glucose levels were related to an increased risk of dementia in both participants with or without diabetes. However, neither insulin levels nor function were assessed in this study. A similar conclusion has been drawn from the rodent study by Ramos-Rodriguez et al. (Ramos-Rodriguez et al., 2013). In this study, db/db mice with hyperglycemia develop episodic memory impairments, whereas C57 mice fed a HFD for 18 weeks did not develop hyperglycemia nor memory impairments. For both strains of mice, insulin levels were significantly increased compared with control mice. In contrast, the present study shows that, with hyperinsulinemia but different blood glucose levels, after 6 to 8 months of HFD one strain (C57) develops peripheral neuropathy whereas the other (Swiss Webster) develops central neuropathy. Although STZ-diabetic and HFD-fed C57 mice displayed significant hyperglycemia, HFD-fed C57 mice were not overtly hyperglycemic. Hyperglycemia might, therefore, contribute to the peripheral neuropathy; however, our results suggest a limited contribution of hyperglycemia in the development of neuropathy, whereas dysregulation of insulin signaling, associated with other parameters such as dyslipidemia or low plasma adiponectin levels, might contribute to diabetic and pre-diabetic peripheral and central neuropathy, respectively. Indeed, several human studies have shown that patients diagnosed with pre-diabetes or metabolic syndrome develop peripheral neuropathy before overt hyperglycemia (Papanas and Ziegler, 2012; Sahin et al., 2008), supporting the limited role of hyperglycemia in the development of neuropathy. In addition, epidemiological studies support the role of insulin resistance as a risk factor for central-neuropathy-associated cognitive deficits or AD (reviewed in Cholerton et al., 2013; McNay and Recknagel, 2011). Delivery of insulin to the hippocampus of rats enhances spatial memory via a PI3K-dependent manner and insulin resistance in HFD-fed rats impairs cognitive functions, showing that insulin and its functional pathway are required for optimal hippocampal memory processing (McNay et al., 2010). Similarly, whole body insulin sensitivity was associated with impairments in behavioral flexibility in insulin-resistant rats that were not frankly diabetic, suggesting that insulin resistance affects cognitive ability and this precedes the development of diabetes (McNeilly et al., 2011).

The current study supports the role of insulin and its impairments in the development of neuropathy and underlines that other parameters, in addition to insulin deficiency or resistance, might predispose the PNS and/or the CNS to damage. For CNS damage, in addition to a decreased insulin-signaling pathway associated with an increase of GSK3 activity, phosphorylated tau and Aβ levels, and a decrease in synaptophysin protein levels in the brain, the factor differentiating Swiss Webster from C57 mice fed a HFD is decreased plasma adiponectin levels. C57 mice fed a HFD have similar plasma and brain adiponectin levels and do not develop learning and memory deficits even with a long duration of obesity/type 2 diabetes. This is in agreement with the lack of cognitive deficits in C57 fed a HFD observed by Ramos-Rodriguez et al. (Ramos-Rodriguez et al., 2013) and in HFD-fed F344 rats (Pancani et al., 2013). Adiponectin, an adipokine secreted by adipose tissue with insulin-sensitizing, anti-inflammatory and antioxidant properties, has three isoforms, with the HMW isoform considered to be the major active protein (Kobayashi et al., 2004; Lara-Castro et al., 2006). Adiponectin can modulate the vascular response to lipid and inflammatory reaction: adiponectin levels were found to be significantly decreased in stroke patients with central microbleeds compared with stroke patients without microbleeds (Huang et al., 2013; Okamoto et al., 2006). Circulating levels of adiponectin are inversely correlated to obesity/type 2 diabetes (Ahima, 2006). However, adiponectin, total or HMW, levels did not correlate with peripheral neuropathy in type 2 diabetic patients (Kato et al., 2008; Matsuda et al., 2004). In contrast, adipokines play a role in the brain and are involved in the modulation of certain cognitive functions (Gustafson, 2012). Although somewhat contradictory, several recent studies have shown changes in adiponectin levels that are associated with dementia: an increased adiponectin level in women was identified as a risk factor for dementia and AD (van Himbergen et al., 2012). Another study did not find any significant difference between individuals with AD and control subjects (Warren et al., 2012), whereas others found that serum levels of adiponectin were significantly lower in individuals with mild cognitive impairment (MCI) or AD compared with control subjects (Teixeira et al., 2013). In addition, in AD patients, donepezil, one of the currently approved treatments for AD, restored plasma adiponectin levels (Pákáski et al., 2014). In our study, adiponectin levels were significantly decreased in the plasma of Swiss Webster mice but not in C57 mice that did not experience learning deficits. This decrease in plasma levels was counterbalanced by a close-to-significant increase of adiponectin and adiponectin receptor protein levels in the hippocampus of Swiss Webster mice fed a HFD. These data are consistent with the decrease of plasma adiponectin levels but increased immunoreactivity to adiponectin and adiponectin receptor in the cortex and hippocampus of rats after brain injury (Takeuchi et al., 2013). In cell culture, adiponectin was shown to protect against Aβ neurotoxicity (Chan et al., 2012). These reports suggest that adiponectin might participate in protective mechanisms in the brain and therefore increased adiponectin levels in the brain might be a compensatory mechanism that contributes to a decrease in plasma levels. In the diabetic brain, the decrease in plasma adiponectin levels might result from an upregulation of brain adiponectin and its signaling to protect against Aβ neurotoxicity. In association with insulin-signaling impairment, peripheral adiponectin level could be a marker of central neuropathy.

The other contributing parameters or mechanisms that might determine the phenotype of the neuropathy are fat metabolism and lipid dysregulation. C57, but not Swiss Webster, mice fed a HFD for 32 weeks developed peripheral neuropathy with nerve conduction velocity slowing, consistent with others (Obrosova et al., 2007; Vincent et al., 2009), and thermal hyperalgesia; symptoms also seen in patients (Baron et al., 2009) and HFD and type 2 diabetic rodents (Groover et al., 2013; Kamiya et al., 2005; Piercy et al., 1999). C57 mice also develop a significant increase in plasma TGs and cholesterol levels, whereas no changes in levels were observed for Swiss Webster fed a HFD. Few publications exist involving Swiss Webster mice fed a HFD, whereas numerous studies show decreased nerve conduction velocity, increased levels of cholesterol and TGs, and moderate hyperglycemia in C57 mice fed a HFD (Obrosova et al., 2007; Vincent et al., 2009; Watcho et al., 2010) as observed here. C57 mice fed a HFD have increased TG levels but no detectable alteration of level or distribution of APP, Aβ, phosphorylated tau (Moroz et al., 2008) or synaptophysin in the brain and no water maze deficits after 5 and 10 months on HFD (Mielke et al., 2006). These studies support our demonstration that dyslipidemia can predispose to peripheral but not central diabetic neuropathy. Our data correlate with human studies showing that individuals with diabetes have increased TG and low-density lipoprotein (LDL) cholesterol and decreased high-density lipoprotein (HDL) cholesterol levels, and this correlates with diabetic peripheral neuropathy (Clemens et al., 2004; Wiggin et al., 2009). Hypertriglyceridemia has been shown as an important risk factor for amputation in individuals with diabetes (Callaghan et al., 2011) and dyslipidemia has been proposed as an important risk factor for diabetic neuropathy (Ansquer et al., 2009; Vincent et al., 2009).

This study, using two strains of mice fed a HFD, allowed a separation of the role of glucose and insulin in the development of neuropathy and points towards possible parameters that determine the phenotype of the neuropathy associated with diabetes and/or obesity. During metabolic syndrome (pre-diabetes), in addition to peripheral insulin dysregulation, lipid metabolism might determine the development of peripheral neuropathy, and an impaired brain insulin pathway, increased levels of tau and decreased adiponectin levels might predispose to central neuropathy associated with cognitive deficits. STZ-induced insulin-deficient diabetes in those two strains of mice support these findings because STZ-diabetic C57 and Swiss Webster (Jolivalt et al., 2008) mice displayed both high levels of TGs [this report and Kim and Kim (Kim and Kim, 2012), respectively] and low levels of plasma adiponectin, and develop both PNS and CNS deficits. The two models used here will allow testing of therapeutic strategies depending on the type of neuropathy. These strategies could include good insulin management with insulin and/or insulin sensitizer in addition to lipid profile control and restoration of adiponectin level to prevent the development and progression of PNS and CNS diabetic complications. Further detailed studies of the differences between the two models will help understand the mechanisms governing both peripheral and central neuropathy.

## MATERIALS AND METHODS

### Animals and diet

Adult female Swiss Webster and C57BL6 (C57) mice were purchased from Harlan Industries (Placentia, CA, USA). Animals were housed four to five per cage with free access to food and water and maintained in a vivarium approved by the American Association for the Accreditation of Laboratory Animal Care. All animal studies were carried out according to protocols approved by the Institutional Animal Care and Use Committee of the University of California San Diego. Ten mice were used per group. Mice were fed a low-fat diet (LFD; 10% kcal from fat, Research Diet Inc., New Brunswick, NJ, USA) or a high-fat diet (HFD; 60% kcal from fat, Research Diet Inc., New Brunswick, NJ, USA) for 32 weeks.

### Induction of type 1 diabetes

Insulin-deficient diabetes was induced in 4-month-old C57BL6 mice following an overnight fast by intraperitoneal (i.p.) injection of streptozotocin (STZ; Sigma, St Louis, MO, USA) at 90 mg/kg body weight dissolved in 0.9% sterile saline, on 2 successive days. Hyperglycemia was confirmed using a strip-operated reflectance meter in a blood sample obtained by tail prick 4 days after STZ injection and in another sample collected at the conclusion of the study.

### Barnes circular maze task

Learning and memory abilities were assessed using the Barnes maze test as described previously (Jolivalt et al., 2010; Jolivalt et al., 2008). Briefly, the Barnes circular maze consists of an illuminated white circular platform with 20 holes (5 cm diameter) equally spaced and located 5 cm from the perimeter. A black escape box was placed under one of the holes. A cue was placed behind the hole with the escape box. The mouse was placed in the middle of the platform and allowed to explore the maze. Timing of the session ended when the mouse found the box or after 5 minutes had elapsed. Mice were tested at 8 and 31 weeks of HFD, once a day, for 5 consecutive days for the learning phase and after 3 days without testing at day 9 for the memory phase of the test.

### Rotarod

Mice were placed on a rotarod (Stoelting Co., Wood Dale, IL, USA) to assess motor coordination and physical condition. The device accelerated from 4 to 40 rpm over 5 minutes. The amount of time spent on the rod before loss of balance was recorded over three trials. All trials were performed on the same day.

### Paw thermal response latency

Mice were placed in an observation chamber on top of the thermal testing apparatus (UARD, San Diego, CA, USA) and allowed to acclimate to the warmed glass surface (30°C) and surroundings for 20 minutes. The mobile heat source was placed below the center of the hind paw and turned on, activating a timer and locally warming the glass surface. Paw withdrawal triggered movement sensors that stopped the timer and turned off the heat source. Both paws were measured and the mean used as a composite score for each mouse. To avoid tissue damage, the upper cut-off limit was set at 20 seconds (Calcutt, 2004).

### Nerve conduction velocity

Under isoflurane anesthesia, core temperature was maintained at 37°C using a heated pad. The sciatic nerve was stimulated with needle electrodes at the sciatic notch or ankle. Using AD Instruments Powerlab 4/30 (AD Instruments, Colorado Springs, CO, USA), a 50 μs stimulus of 6.75 volts was delivered and the duration of conduction from either the sciatic notch or the ankle of the hind paw was recorded from interosseous muscles of the foot with fine-needle electrodes, using the Scope 4 program. The difference in response latencies of the M wave after stimulation at the sciatic notch and ankle was recorded as the time required for the motor nerve conduction between those two sites. The distance between stimulation sites was measured on fully extended hind limb and divided by the M-wave latencies difference to calculate sciatic motor nerve conduction velocity (MNCV).

### Tissue preparation for western blot analysis

Mice were sacrificed by isoflurane and decapitation. Brains were dissected within 1 minute and hippocampi were homogenized in buffer (50 mM Tris-HCl pH 7.4, 150 mM NaCl, 0.5% Triton X, 1 mM EDTA, protease inhibitor cocktail). Homogenates were centrifuged at 13,000 *g* for 30 minutes and supernatants stored in aliquots at −80°C. Protein concentration was assessed using the bicinchoninic acid method (BCA protein assay kit, Pierce, Rockford, IL, USA).

### Western blotting

Aliquots of the hippocampus homogenates were boiled in Laemmli LDS sample buffer (Invitrogen, Carlsbad, CA, USA). Up to 20 μg of total extract protein were separated on 4–12% SDS-PAGE Bis-Tris gels (Novex, Invitrogen, Carlsbad, CA, USA) and immunoblotted on nitrocellulose. To maximize the number of proteins analyzed by western blot, membranes were cut along the molecular markers into strips containing the proteins of interest. Blot strips were incubated with antibodies against phospho-insulin receptor (phosphorylated Ser972, 1/1000, Upstate, Temecula, CA, USA), insulin receptor (1/200, Chemicon International, Temecula, CA, USA), phospho-GSK3β (phospho-Ser9; 1/1000, Cell Signaling Technology, USA), GSK3α/β (1/5000, Chemicon International, Temecula, CA, USA), Aβ (mouse monoclonal, clone 6E10, 1/1000, Covance/Signet Laboratories, Berkeley, CA, USA), phosphorylated tau (Ser202, 1/3000, Chemicon), synaptophysin (1/10,000, Chemicon), adiponectin (1/1000, Abcam, Cambridge, MA, USA), adiponectin receptor 1 (1/1000, Abcam, Cambridge, MA, USA), actin (1/5000, Sigma) or cyclophilin B (1/5000, Abcam, Cambridge, MA, USA) followed by secondary antibodies tagged with infrared dyes (IRDye, 1/15,000, LI-COR Biosciences, Lincoln, NE, USA). Blots were developed using infrared imaging system Odyssey Fc (LI-COR Biosciences, Lincoln, NE, USA). For each animal, band intensities were normalized by calculating the ratio of the intensity of the band corresponding to primary antigen of interest to the intensity of the band corresponding to cyclophilin B.

### Plasma analysis

Plasma obtained at termination of the study was assessed for insulin (Ultrasensitive mouse insulin ELISA, Mercodia AB, Uppsala, Sweden), adiponectin (HMW and total adiponectin ELISA, Alpco Immunoassays, Salem, NH, USA), and for cholesterol and TG levels using the oxygen-rate analyzer GM7 (Analox Instruments, Lunenburg, MA, USA).

### Statistical analysis

Data are presented as group mean + s.e.m. Differences between groups were analyzed using Student’s *t*-test versus their respective control (LFD) or by one-way ANOVA followed by Tukey post-hoc test when comparing STZ mice to LFD and HFD C57 mice.
